# Dual inhibitory action of trazodone on dorsal raphe serotonergic neurons through 5-HT_1A_ receptor partial agonism and α_1_-adrenoceptor antagonism

**DOI:** 10.1371/journal.pone.0222855

**Published:** 2019-09-26

**Authors:** Alberto Montalbano, Boris Mlinar, Francesco Bonfiglio, Lorenzo Polenzani, Maurizio Magnani, Renato Corradetti

**Affiliations:** 1 NEUROFARBA—Dipartimento di Neuroscienze, Psicologia, Area del Farmaco e Salute del Bambino, Università di Firenze, Firenze, Italia; 2 Angelini RR&D (Research, Regulatory & Development), Angelini S.p.A, S.Palomba-Pomezia (Roma), Italia; Indiana University School of Medicine, UNITED STATES

## Abstract

Trazodone is an antidepressant drug with considerable affinity for 5-HT_1A_ receptors and α_1_-adrenoceptors for which the drug is competitive agonist and antagonist, respectively. In this study, we used cell-attached or whole-cell patch-clamp recordings to characterize the effects of trazodone at somatodendritic 5-HT_1A_ receptors (5-HT_1A_ARs) and α_1_-adrenoceptors of serotonergic neurons in rodent dorsal raphe slices. To reveal the effects of trazodone at α_1_-adrenoceptors, the baseline firing of 5-HT neurons was facilitated by applying the selective α_1_-adrenoceptor agonist phenylephrine at various concentrations. In the absence of phenylephrine, trazodone (1–10 μM) concentration-dependently silenced neurons through activation of 5-HT_1A_ARs. The effect was fully antagonized by the selective 5-HT_1A_ receptor antagonist Way-100635. With 5-HT_1A_ receptors blocked by Way-100635, trazodone (1–10 μM) concentration-dependently inhibited neuron firing facilitated by 1 μM phenylephrine. Parallel rightward shift of dose-response curves for trazodone recorded in higher phenylephrine concentrations (10–100 μM) indicated competitive antagonism at α_1_-adrenoceptors. Both effects of trazodone were also observed in slices from *Tph2*^*-/-*^ mice that lack synthesis of brain serotonin, showing that the activation of 5-HT_1A_ARs was not mediated by endogenous serotonin. In whole-cell recordings, trazodone activated 5-HT_1A_AR-coupled G protein-activated inwardly-rectifying (GIRK) channel conductance with weak partial agonist efficacy (~35%) compared to that of the full agonist 5-CT. Collectively our data show that trazodone, at concentrations relevant to its clinical effects, exerts weak partial agonism at 5-HT_1A_ARs and disfacilitation of firing through α_1_-adrenoceptor antagonism. These two actions converge in inhibiting dorsal raphe serotonergic neuron activity, albeit with varying contribution depending on the intensity of α_1_-adrenoceptor stimulation.

## Introduction

The brain serotonin (5-HT) system modulates a variety of brain functions including mood, cognition, emotional behaviour, and sleep [1, 2, 3] and its dysregulation appears to contribute in related psychopathological states such as depression, anxiety, impulsivity and aggression [4].

Trazodone is an antidepressant drug that, in addition to its inhibitory activity at cell membrane 5-HT transporter (SERT), is a competitive ligand at 5-HT_1A_ 5-HT_2A_, 5-HT_2C_ receptors and α_1_-adrenoceptors for which it displays considerable affinity [5, 6]. Although these pharmacological properties have been suggested to contribute a favourable safety profile such as facilitation of sleep and reduced sexual dysfunction [7], the direct effects of trazodone on serotoninergic neuron activity are still not adequately known for modelling the possible pharmacological mechanisms underlying the therapeutic action(s) of the drug.

In the dorsal raphe nucleus (DRN) the spontaneous activity of serotonergic neurons is maximally facilitated during wake by noradrenergic input via α_1_-adrenoceptor stimulation [8] and activation of 5-HT system contributes to arousal [9, 10]. Under these conditions, the firing of serotonergic neurons is tonically limited *via* the stimulation of somatodendritic 5-HT_1A_ autoreceptors (5-HT_1A_ARs) exerted by the endogenous 5-HT present in the extracellular space surrounding serotonergic neurons [11, 12]. However, increased extracellular 5-HT in raphe nuclei acutely produced by block of SERT results in (auto)inhibition of serotonergic neuron activity and consequently in reduction of 5-HT release in projection brain areas. This is believed to delay the onset of antidepressant therapeutic effect until subsensitivity of 5-HT1AARs develops to weaken the autoinhibitory feedback [13, 14]. On the basis of this notion it has been proposed that the association of 5 HT_1A_ receptor antagonists could hasten the response to monoamine uptake blockers [15, 16, 17] and, more recently, that antidepressant drugs with partial agonist property at 5 HT_1A_ receptors could display faster onset of therapeutic response [18].

*In vivo* recording of serotonergic neuron activity in anaesthetized rats showed that acute administration of trazodone inhibits DRN serotonergic neuron firing [19] an action mediated by activation of somatodendritic 5-HT_1A_ARs which desensitize after chronic treatment with the antidepressant drug [20]. Nevertheless, the direct functional effects of trazodone on 5-HT_1A_ARs are still insufficiently characterized to establish whether the drug exerts full or partial agonism at 5-HT_1A_ARs. In the rat, in a functional assay *in vitro* trazodone was found to activate [^35^S]GTPγS binding with weak efficacy [21], while *in vivo* its action on serotonergic neuron firing appeared consistent with stronger agonism at 5-HT_1A_ARs [22].

The interplay between the 5-HT_1A_ receptor agonist activity and α_1_-adrenoceptor antagonist activity of trazodone at the level of serotonergic neurons likely plays a crucial role in regulating the firing of serotonergic neurons, hence the release of 5-HT in projection areas during acute and chronic administration of the drug. Actually, a strong noradrenergic input to the dorsal and other raphe nuclei has been identified [23, 24] which tonically activates raphe serotonergic neurons through postsynaptic α_1_-adrenoceptors [25]. Therefore, the α_1_-adrenoceptor antagonist properties of trazodone could participate to the inhibiton of DRN serotonergic neuron firing by reducing the noradrenergic drive, but experiments *in vivo* were unable to quantify this action in the DRN in conditions in which trazodone appeared to have α_1_-adrenoceptor blocking effects in the hippocampus [22].

In the present work we designed *in vitro* experiments directed to quantify the agonist efficacy of trazodone at 5-HT_1A_ARs of DRN serotonergic neurons and to establish the possible effect of α_1_-adrenoceptor antagonism. Here we show that trazodone exerts weak partial agonist action at 5-HT_1A_ARs and α_1_-adrenoceptor antagonism at low micromolar concentrations that are relevant to the therapeutic effects of the drugs.

## Materials and methods

### Animals and animal care

Animal care and experimental procedures strictly complied with the European Communities Council Directive (2010/63/UE) and were approved by the Italian Ministry of Health (Aut: 224/2017-PR and 938/2017-PR). Every effort was made to reduce the number of animals used. Male Wistar rats were purchased from Envigo Italy (Milan, Italy). Tryptophan hydroxylase-2 knock-out (*Tph2*^*-/-*^) mice were obtained from Prof. K.P. Lesch (University of Würzburg, Würzburg, Germany). Animals were housed on a 12:12 h day-night cycle with food and water *ad libitum*.

Procedures for tissue isolation, slice superfusion and electrophysiological recording from DRN serotonergic neurons of rat and mouse have been previously described in detail [26, 27, 28].

### Preparation of brain slices

Slices were prepared from animals aged 4–10 weeks (4–5 weeks for whole-cell patch-clamp recordings). Animals were deeply anesthetized with isoflurane and decapitated. Brains were rapidly removed and dissected in ice-cold gassed (95% O_2_ and 5% CO_2_) artificial cerebrospinal fluid (ACSF) composed of: 124 mM NaCl, 2.75 mM KCl, 1.25 mM NaH_2_PO_4_, 1.3 mM MgCl_2_, 2 mM CaCl_2_, 26 mM NaHCO_3_, 11 mM D-glucose. The brainstem was sliced coronally into 200 μm thick slices with a vibratome (DSK, T1000, Dosaka, Japan). After recovery for at least 90 min at room temperature, the slices were individually transferred to the recording chamber and superfused continuously, at a rate of 2 ml min^-1^, with warmed ACSF (Warner Instruments in-line heater TC324-C). Slices were allowed to equilibrate for at least 15 min before the beginning of the recording. Drugs were bath-applied through a peristaltic pump-driven perfusion system and a complete exchange of the recording chamber volume occurred in approximately 1 min. Neurons within DRN were visualized by infrared differential interference contrast (IR-DIC) video microscopy with a Newicon camera (C2400-07; Hamamatsu, Hamamatsu City, Japan) mounted on an upright microscope (Axioskop; Zeiss, Göttingen, Germany). Recordings were made using an EPC-10 amplifier (HEKA Elektronic, Lamberecht, Germany). Patch pipettes were prepared from thick-walled borosilicate glass on a P-97 Brown-Flaming electrode puller (Sutter Instruments, Novato, CA, USA). Data were analyzed using Patchmaster 2 (HEKA Elektronic), Clampfit 9.2 (Molecular Devices, Sunnyvale, CA, USA) and Prism 7 software (GraphPad Software, San Diego, CA, USA).

### Electrophysiology

Action potential firing activity of serotonergic neurons was recorded by loose-seal cell-attached recordings at a temperature of 34–36°C. To reproduce in slices the noradrenergic drive which facilitates serotonergic neuron firing during wakefulness [8]), ACSF was supplemented with the α_1_-adrenoceptor agonist phenylephrine (PE, 10 μM, unless otherwise stated). In a set of experiments neuron firing was facilitated in an α_1_-adrenoceptor–independent mode using a modified ACSF containing low Ca^2+^ (0.67 mM), high K^+^ (5.5 mM) and no PE. When appropriate, ACSF contained a “cocktail” of glutamate GABA/glycine and adenosine A_1_ receptor blockers consisting of: 10 μM NBQX (2,3-dioxo-6-nitro-1,2,3,4-tetrahydrobenzo[f]quinoxaline-7-sulfonamide disodium salt), 20 μM d-AP5 (d-(-)-2-amino-5-phosphonopentanoic acid), 10 μM SR-95531 (6-imino-3-(4-methoxyphenyl)-1(6H)-pyridazinebutanoic acid hydrobromide), 2 μM CGP-55845 (3-N[1-(S)-(3,4-dichlorophenyl)ethyl]amino-2-(S)-hydroxypropyl-P-benzyl-phosphinic acid hydrochloride) and 10 μM strychnine hydrochloride, 0.2 μM DPCPX (8-Cyclopentyl-1,3-dipropylxanthine) to functionally isolate the recorded neuron from major synaptic input. Patch pipettes were prepared from thick-walled borosilicate glass on a P-97 Brown-Flaming electrode puller (Sutter Instruments, Novato, CA, USA) and had resistances of 3–6 MΩ when filled with solution containing (in mM): 125 NaCl, 10 HEPES, 2.75 KCl, 2 CaCl_2_, 1.3 MgCl_2_ (pH 7.4 with NaOH). Loose-seal cell-attached recordings (5–20 MΩ seal resistance) were acquired continuously in the voltage-clamp mode. Signals were filtered at 3 kHz and digitized at 10 kHz. The firing rate was reported using 10 s bins. Data were analysed using Clampfit 9.2 (Molecular Devices, Sunnyvale, CA, USA). Most recorded neurons were located in the dorsal and ventromedial part of the DRN. Neurons were identified according to electrophysiological criteria [12, 29]. Only neurons with a stable baseline firing rate were used.

Methods used for measuring G protein-activated inwardly-rectifying (GIRK) channel conductance have been detailed previously [12, 27]. All experiments were performed in ACSF supplemented with a cocktail of neurotransmitter blockers (*see* above). ACSF contained 5.5 mM K^+^ to increase the driving force for inward potassium current (the additional 2.75 mM by Na^+^ substitution). Recording pipettes had a resistance of 2–5 MΩ. The pipette solution consisted of: 120 mM K-gluconate, 15 mM KCl, 2 mM MgCl_2_, 10 mM HEPES, 0.1 mM EGTA, 10 mM Na_2_phosphocreatine, 4 mM MgATP, 0.3 mM Na_3_GTP (pH 7.35 with KOH). After establishing whole-cell recording configuration, serotonergic neurons were identified on the basis of electrophysiological properties displayed in current-clamp mode (action potential half-height width > 1.1 ms; absent or very small fast afterhyperpolarization; sustained repetitive firing in response to depolarizing current injection). In current-clamp recordings signals were filtered at 5 kHz and digitized at 25 kHz. To estimate inwardly-rectifying K^+^ conductance, we used hyperpolarizing voltage ramps from the holding potential of -65 mV (to -125 mV, every 10 s; 100 mV s^-1^; 3 kHz cutoff frequency low-pass filter; 10 kHz sampling frequency) and measured the conductance from the slope of inward K^+^ current in the range from -110 to -85 mV (G_-110/-85 mV_). To monitor access resistance throughout the recording, hyperpolarizing pulses (10 mV; 100 ms duration; 16 kHz low-pass filter; 25 kHz sampling frequency; cell capacitance cancellation circuit switched off) were interlaced with ramps. Access resistance was not compensated and when it was higher than 25 MΩ recordings were discarded. Concentration–response curves for trazodone were obtained using a cumulative protocol in which increasing concentrations were applied in 7–10 min intervals as appropriate. Average of 7 consecutive individual ramps, corresponding to the segment with the maximal effect for the given trazodone concentration were used to obtain conductance values. To calculate net 5-HT_1A_AR-activated GIRK current and conductance (G_5-HT1A_) present during a recording, the 5-HT_1A_AR-insensitive current was measured at the end of a recording following application of the selective 5-HT_1A_ receptor antagonist Way-100635 (20 nM; 10–15 min) and subtracted from the total current.

### Calculation of concentration-response relationships

For creating cumulative concentration-response curves for trazodone, the drug was applied for 10–30 min (as appropriate for the specific experimental objective) and mean firing rates were calculated from the last one-minute segment of each experimental epoch (e.g. baseline, trazodone 0.3 μM, 1 μM, etc.). Trazodone dose-response relationship for suppression of serotonergic neuron firing was computed by fitting data to the logistic equation *b + (a—b) / (1 + (EC*_*50*_ */ [trazodone])*^*n*^_*H*_*)*, where *EC*_*50*_ is the half-maximally effective concentration, *n*_*H*_ is the Hill coefficient, a is the baseline firing rate and *b* is the fraction remaining at the maximal trazodone effect. Fitting of average dose-response relationship of normalized firing rate: for each experiment, firing rate was measured during the last 2 min of drug application for each concentration and normalized by taking the pre-drug baseline firing rate as unity. Then the relationship for suppression of serotonergic neuron firing was computed by fitting mean values (± SD) for each concentration to the logistic equation, *b + (1—b) / (1 + (EC*_*50*_ */ [trazodone])*^*n*^_*H*_*)*, where *EC*_*50*_ is the half-maximally effective concentration, *n*_*H*_ is the Hill coefficient and *b* is the fraction remaining at the maximal trazodone effect.

The respective fractional occupancy of 5-HT_1A_ receptors by trazodone and the full agonist, either 5-carboxamidotryptamine (5-CT) or 5-HT, in the presence of trazodone was calculated using the Gaddum equation: *[AR]/Rt = [A]/([A]+Ka(1+[B]/Kb))*, where [AR]/Rt is the fractional occupancy by the full agonist, [A] is the concentration of the full agonist with Ka affinity constant and [B] and Kb are trazodone concentration and affinity, respectively. To allow comparison of theoretical values with the responses experimentally obtained in rat serotonergic neurons, we calculated the occupancies for 5-CT and trazodone by applying the K_i_ of the two agonists for the rodent 5-HT_1A_ (5-CT: K_i_ = 0.325 nM, [30]; trazodone: K_i_ = 42 nM, [5]). For the action of trazodone on 5-HT we used the K_i_ of the two agonists for the human 5-HT_1A_ receptor (5-HT: K_i_ = 3.98 nM, [31, 32]; trazodone: K_i_ = 96 nM, [6]) to allow inference on the effects of trazodone on responses to endogenous 5-HT *in vivo*.

In both cases, the expected total activation of GIRK conductance was calculated from the respective occupancies of the two compounds by using the measured maximal efficacy of 0.35 for trazodone and 1.0 for the full agonist.

### Drugs

Trazodone was provided by A.C.R.A.F. S.p.A. (Santa Palomba, Rome). 5-HT, SR-95531, *d*-AP5, NBQX were purchased from Ascent Scientific Ltd. (Bristol, UK). Way-100635 maleate, CGP-55845 hydrochloride, 5-CT and DPCPX were purchased from Tocris Bioscience (Bristol, UK). L-Phenylephrine and strychnine hydrochloride from Sigma-Aldrich S.r.l. (Milan, Italy).

### Statistical analysis

Data are presented as geometric mean and confidence interval (C.I.) or mean ± SD as appropriate. Statistical comparisons were performed by ANOVA followed by Tukey *post-hoc* test, paired or unpaired t-test (two tailed), as appropriate. Pearson test was used to assess for correlation between variables. All the statistical tests were performed by GraphPad Prism version 7. Throughout the analyses, statistical significance was taken as p < 0.05.

## Results

### Concentration-dependent inhibition of serotonergic neuron firing by trazodone is mediated by more than one action

**Fig 1:**
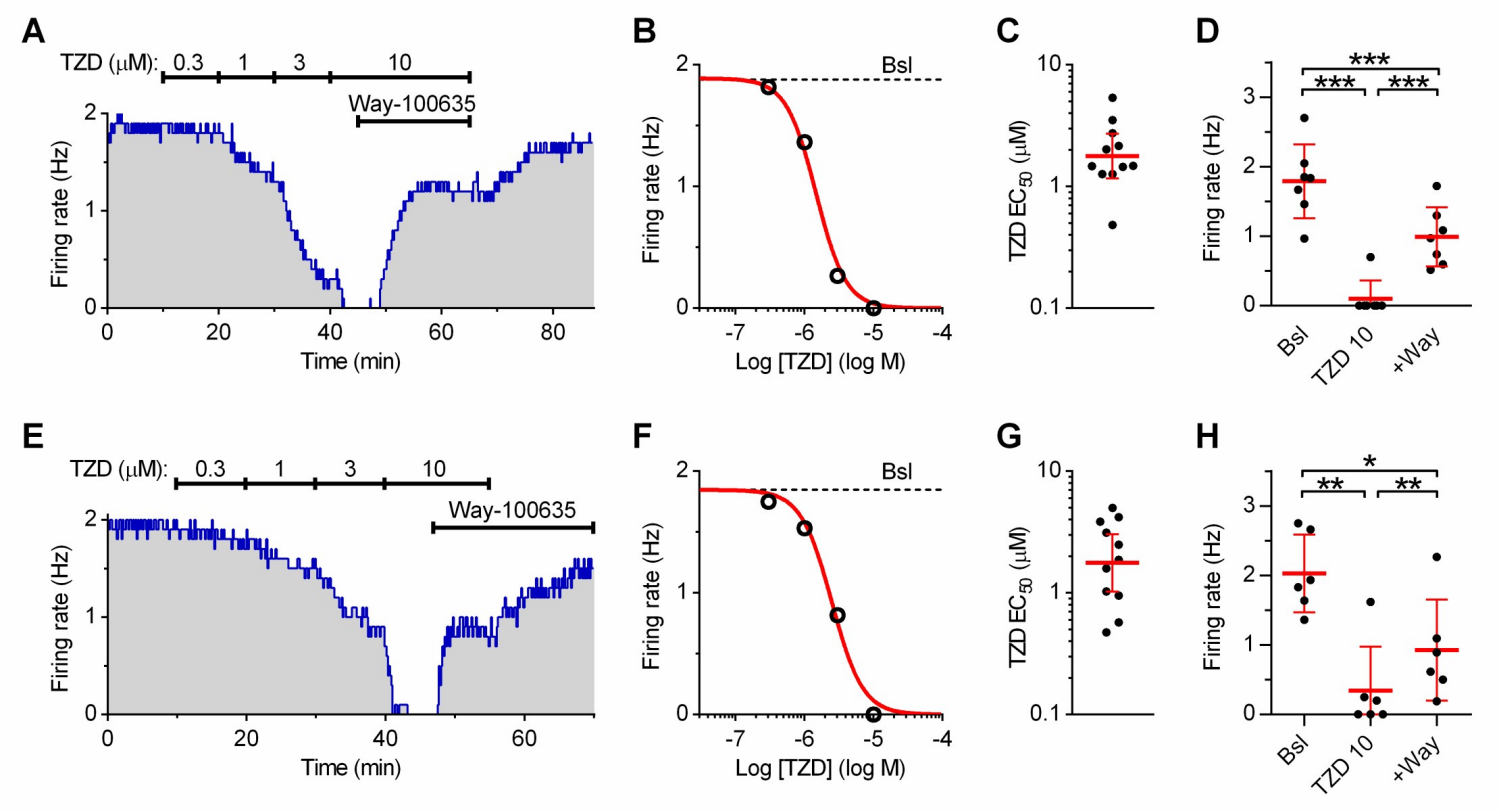
The inhibition of 5-HT neuron activity by trazodone. **(A-D)**: Effect of trazodone (TZD) in control ACSF. **(A)** Representative recording illustrating the time-course of the changes in firing frequency produced by increasing concentrations of trazodone. The maximal effect was partially reversed by the selective 5-HT_1A_ receptor antagonist Way-100635 (20 nM). **(B)** Dose-response curve (DRC) resulting from the experiment shown in **(A)**. Red line is the best least-squares fit to the logistic equation. **(C)** Scatter plot of the individual EC_50_ values calculated from all experiments (n = 11). **(D)** Antagonism of trazodone (10 μM) effect by addition of Way-100635 (+Way; n = 7; One-way RM ANOVA: F_(2,6)_ = 107.6; p <0.0001) in the subset of experiments in which Way was added at the end of the DRC, e.g. in (A). **(E-H)**: Effect of trazodone in a cocktail of antagonists (*see* methods). **(E)** Representative recording of the concentration-dependent changes in firing frequency produced by trazodone. The effect of 10 μM trazodone was partially reversed by Way-100635 (20 nM). Note the recovery of firing rate in Way-100635 after the washout of trazodone. **(F)** DRC resulting from the experiment shown in **(E)**. Red line is the best least-squares fit to the logistic equation. **(G)** Scatter plot of the individual EC_50_ values calculated from all experiments (n = 11). **(H)** Antagonism of trazodone (10 μM) effect by Way-100635 (+Way; n = 6; One-way RM ANOVA: F_(2,5)_ = 28.68; p <0.0014) in the subset of experiments in which Way was added at the end of the DRC, e.g. in (A). In **(B)** and **(F)**, dotted line indicates the baseline (Bsl) firing rate before application of trazodone. In **(C)** and **(G)**, red line and error bars indicate the geometric mean and 95% C.I. In all experiments the activity of serotonergic neurons was facilitated by the presence of 10 μM phenylephrine. * p < 0.05; ** p < 0.01; *** p < 0.001 (One-way RM ANOVA, followed by a Tukey *post-hoc* test).

Application of trazodone produced a concentration-dependent inhibition of DRN serotonergic neuron activity facilitated by 10 μM PE in standard ACSF (Fig 1A and 1B).

The effect of trazodone was partially antagonized by the selective 5-HT_1A_ receptor antagonist Way-100635. Trazodone silenced neurons with a mean of EC_50_ values = 1.78 μM (Geometric mean, GM, 95% Confidence Interval, 95% C.I.: 1.165–2.713, n = 11; Fig 1C). In a subset of experiments in which Way-100635 (20 nM) was added to 10 μM trazodone, the firing rate recovered to less of 50% relative to baseline firing rate (47.54 ± 21; 41%; n = 7; Fig 1D) suggesting that trazodone effect was only in part mediated by activation of 5-HT_1A_ARs.

The non 5-HT1AAR-mediated decrease in firing of serotonergic neurons produced by trazodone could indirectly be elicited by changes in the release of GABA, glycine or adenosine from neighbour neurons, or glutamate from terminals. We therefore carried out a set of experiments in ACSF containing a “cocktail” of receptor antagonists for major neurotransmitters (*see* methods).

Under these conditions, the inhibitory effect of trazodone was very similar to that exerted in standard ACSF (Fig 1E–1H). The average EC_50_ value was 1.77 μM (GM, 95% C.I.: 1.023–3.034, n = 11). Statistical comparison of EC_50_ values in individual neurons revealed no differences of trazodone effect in standard ACSF and cocktail ACSF (unpaired t test, two tails: t = 0.2813; df = 20; p = 0.7814). Similar to the previous set of experiments, in a subset of experiments in which Way-100635 (20 nM) was added to 10 μM trazodone, the firing rate recovered to 45.93 ± 30.32% (n = 6, Fig 1H) of baseline firing rate. These results demonstrate that the Way-100635-insensitive inhibitory effect trazodone was directly exerted on serotonergic neurons and not mediated by release of GABA, glycine, adenosine and glutamate from the local network of neurons and terminals. Nevertheless, all subsequent experiments were performed in the presence of synaptic antagonists.

### Trazodone directly activates 5-HT_1A_ receptors in Tph2^-/-^ mice

**Fig 2:**
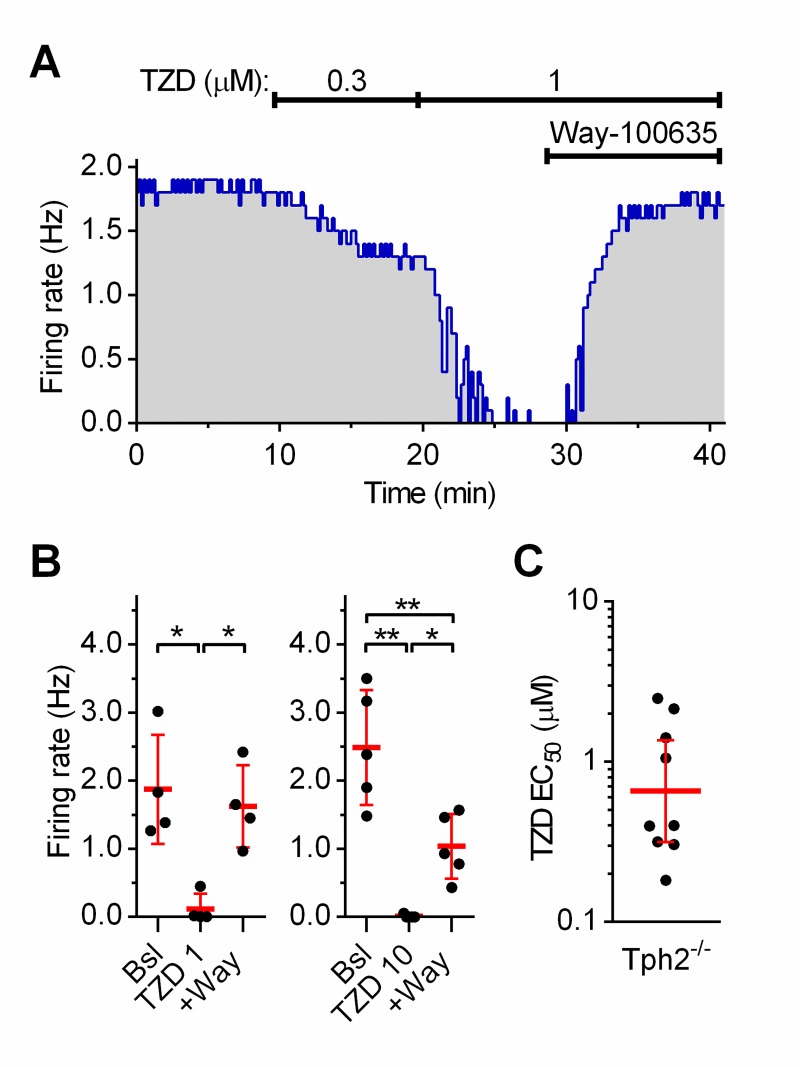
Effect of trazodone in *Tph2*^*-/-*^ mice. **(A)** Representative recording of concentration-dependent effect of trazodone (TZD). The effect was fully antagonized by 20 nM Way-100635. **(B)** Summary of the antagonism exerted by 20 nM Way-100635 (+Way) on responses to 1 μM (n = 4; One-way RM ANOVA: F_(2,3)_ = 36.24; p <0.0045) and 10 μM (n = 5; One-way RM ANOVA: F_(2,4)_ = 41,95; p <0.0028) trazodone. Note the partial antagonism of responses to 10 μM trazodone. **(C)** Scatter plot of the individual EC_50_ values calculated from all experiments (n = 9). Red line and error bars indicate the geometric mean and 95% C.I.. The activity of serotonergic neurons was facilitated by the presence of 10 μM PE. * p < 0.05; ** p < 0.01 (One-way RM ANOVA, followed by a Tukey *post-hoc* test).

To ascertain whether the inhibition of serotonergic neuron firing by trazodone was due to direct stimulation of 5-HT_1A_ARs or was indirectly mediated by a possible raise in extracellular 5-HT caused by SERT inhibition produced by the drug, we repeated these experiments in *Tph2*^*-/-*^ mice that lack synthesis of brain 5-HT [33]). In this set of experiments 20 nM Way-100635 was added to the trazodone concentration that produced more than 90% inhibition of neuron firing (Fig 2A).

In the nine neurons tested, serotonergic neuron firing was silenced at concentrations ≤ 10 μM with an average EC_50_ value of 0.66 μM (GM, 95% C.I.: 0.3157–1.364, n = 9; Fig 2C). As shown in Fig 2A and 2B, in four neurons in which 1 μM trazodone decreased the firing by 96.1 ± 7.3%, the effect was fully antagonized by Way-100635. When 10 μM trazodone was required to silence the recorded neurons, Way-100635 significantly, but only partially, antagonized the effect that recovered by 40.13 ± 6.74% (n = 5, Fig 2B). These results confirmed that trazodone was a direct agonist at 5-HT_1A_ARs. The greater potency of trazodone in inhibiting firing (EC_50_ = 0.66 μM) compared to that observed in rat (EC_50_ = 1.77 μM) is likely to be ascribed to the supersensitivity of 5-HT_1A_ARs in *Tph2*^-/-^ mice compared to *Tph2*^+/+^ mice (~2,5 folds) [34].

### The non 5-HT_1A_ receptor-mediated effect of trazodone depends on α_1_-adrenoceptor activation and is competitively surmounted by the α_1_-adrenoceptor agonist phenylephrine

The experiments reported in 3.1 and 3.2 show that trazodone inhibits the firing of serotonergic neurons by direct activation of 5-HT_1A_ARs and that an additional inhibitory effect of the drug contributes in silencing firing. As trazodone is an α_1_-adrenoceptor ligand and in our experiments the activity of serotonergic neurons was facilitated by the presence of 10 μM PE, the non 5-HT_1A_ receptor-mediated inhibitory effect of trazodone could result from disfacilitation of neuron firing produced by α_1_-adrenoceptor antagonism.

**Fig 3:**
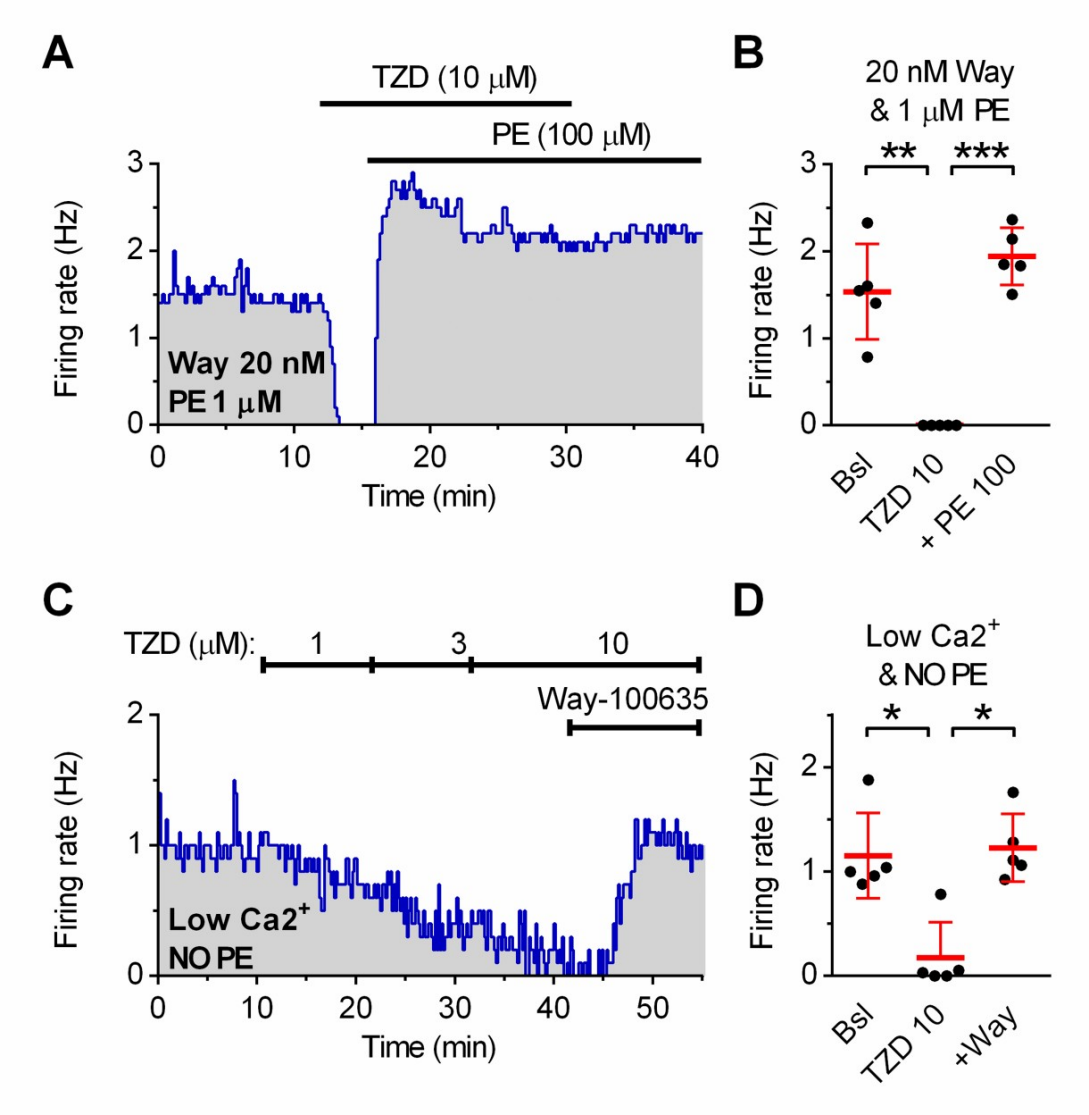
Antagonism at α_1_-adrenoceptors by trazodone contributes in the inhibition of serotonergic neuron activity. **(A)** Representative recording of neuron activity facilitated by 1 μM phenylephrine (PE) in the presence of Way-100635 (+Way; 20 nM). The inhibitory effect of 10 μM trazodone (TZD) is surmounted by addition of 100 μM phenylephrine (PE). **(B)** Summary of five experiments (mean ± SD; One-way RM ANOVA: F_(2,4)_ = 48.74; p <0.0002). **(C)** Representative recording of the concentration-dependent effect of trazodone on neuron activity facilitated by low calcium ACSF (*see* methods) in the absence of PE. **(D)** Summary of five experiments (mean ± SD; One-way RM ANOVA: F_(2,4)_ = 16.21; p <0.0122) * *p*< 0.05; ** *p*< 0.01; *** *p*< 0.001 (One-Way RM ANOVA followed by Tukey *post-hoc* test).

In preparations in which the neuron firing was facilitated by a low concentration of PE (1 μM) in the presence of Way-100635 (20 nM), the application of 10 μM trazodone silenced the neurons and the effect was fully reversed by the addition of 100 μM PE (Fig 3A and 3B; n = 5).

This showed that the non 5-HT_1A_ receptor-mediated effect of trazodone could be surmounted by increasing PE concentration, indicating that trazodone was acting as competitive antagonist at α_1_-adrenoceptors. Accordingly, when the firing was facilitated by low calcium/high potassium ACSF (*see* methods) in the *absence* of PE, trazodone 10 μM produced a pure 5-HT_1A_ receptor-mediated inhibition of serotonergic neuron activity (Fig 3C and 3D).

**Fig 4:**
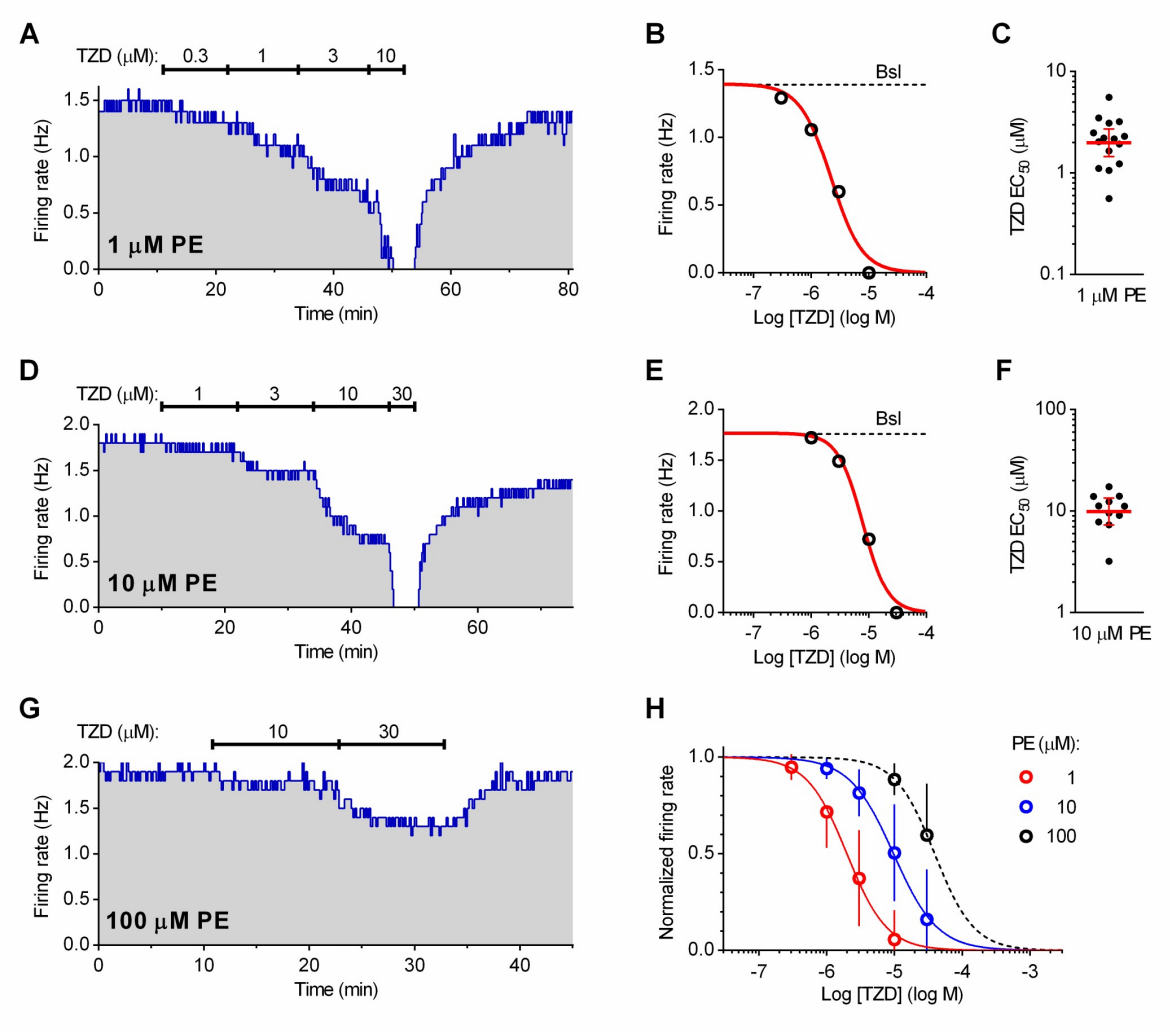
Block of 5-HT_1A_ receptors by Way-100635 uncovers the α_1_-adrenoceptor- dependent effect of trazodone. **(A)** Representative experiment showing the effect of trazodone (TZD) on neuron firing facilitated by 1 μM phenylephrine (PE) in the presence of Way-100635 (20 nM). **(B**) DRC resulting from the experiment shown in **(A)**. Red line is the best least-squares fit to the logistic equation. **(C**) Scatter plot of the individual EC_50_ values calculated from fifteen similar experiments. **(D)** Representative experiment showing the effect of trazodone on neuron firing facilitated by 10 μM PE **(E**) DRC resulting from the experiment shown in **(D)**. Red line is the best least-squares fit to the logistic equation. Note that 30 μM trazodone is required to silence the neuron. **(F**) Scatter plot of the individual EC_50_ values calculated from eleven experiments. **(G)** Representative recording from a set of experiments (n = 9) in which trazodone 10 μM and 30 μM was applied in the presence of 100 μM PE. In **(B)** and **(E)**, dotted line indicates the baseline (Bsl) firing rate before application of trazodone. In **(C)** and **(F)**, red line and error bars indicate the geometric mean and 95% C.I. **(H)** Average DRCs of trazodone effect obtained from the experiments in PE 1–100 μM. Symbols and error bars correspond to the mean values± SD obtained for each concentration in the corresponding set of experiments. Red line is the best least-squares fit to the logistic equation. Data are normalized on average baseline firing rates recorded before trazodone application. The baseline firing rates of neurons recorded in the three groups were (Hz, mean ± SD): 1.840 ± 0.5444 in PE 1μM (n = 15), 1.766 ± 0.4040 in PE 10μM (n = 11) and 1.970 ± 0.5559 in PE 100 μM (n = 9). These values were not significantly different (F_(2.32)_ = 0.4046; p = 0.6706; One-Way ANOVA).

To better characterize the contribution of α_1_-adrenoceptor antagonism in the inhibition of serotonergic neuron firing by trazodone, we compared the concentration-dependent effect of trazodone in preparations where the firing was facilitated by different concentrations of PE (1, 10, 100 μM) in the presence of 20 nM Way-100635 to block the 5-HT_1A_ receptor-mediated effect of trazodone (Fig 4).

In PE 1 μM, trazodone concentration-dependently suppressed neuron firing (Fig 4A and 4B) with an average EC_50_ value of 1.985 μM (GM, 95% C.I.: 1.451–2.716, n = 15; Fig 4C). When neurons were activated by PE 10 μM the average EC_50_ value was increased to 9.881 μM (GM, 95% C.I.: 7.284–13.40, n = 11; Fig 4D–4F). As illustrated in Fig 4G, in PE 100 μM we applied only 10 and 30 μM trazodone to avoid a likely contamination of responses by aspecific effects of the drug at higher concentrations. From this set of experiments (n = 9) individual EC_50_ values could not be calculated, while the mean effects for these two drug concentrations were sufficient to obtain the EC_50_ value for trazodone effect in 100 μM PE (39.07 μM) from the calculated average concentration-response curve. Fig 4H illustrates the rightward shift of concentration-response curves for the α_1_-adrenoceptor-mediated inhibitory effect of trazodone produced by increasing the concentration of PE from 1 μM to 100 μM.

**Fig 5:**
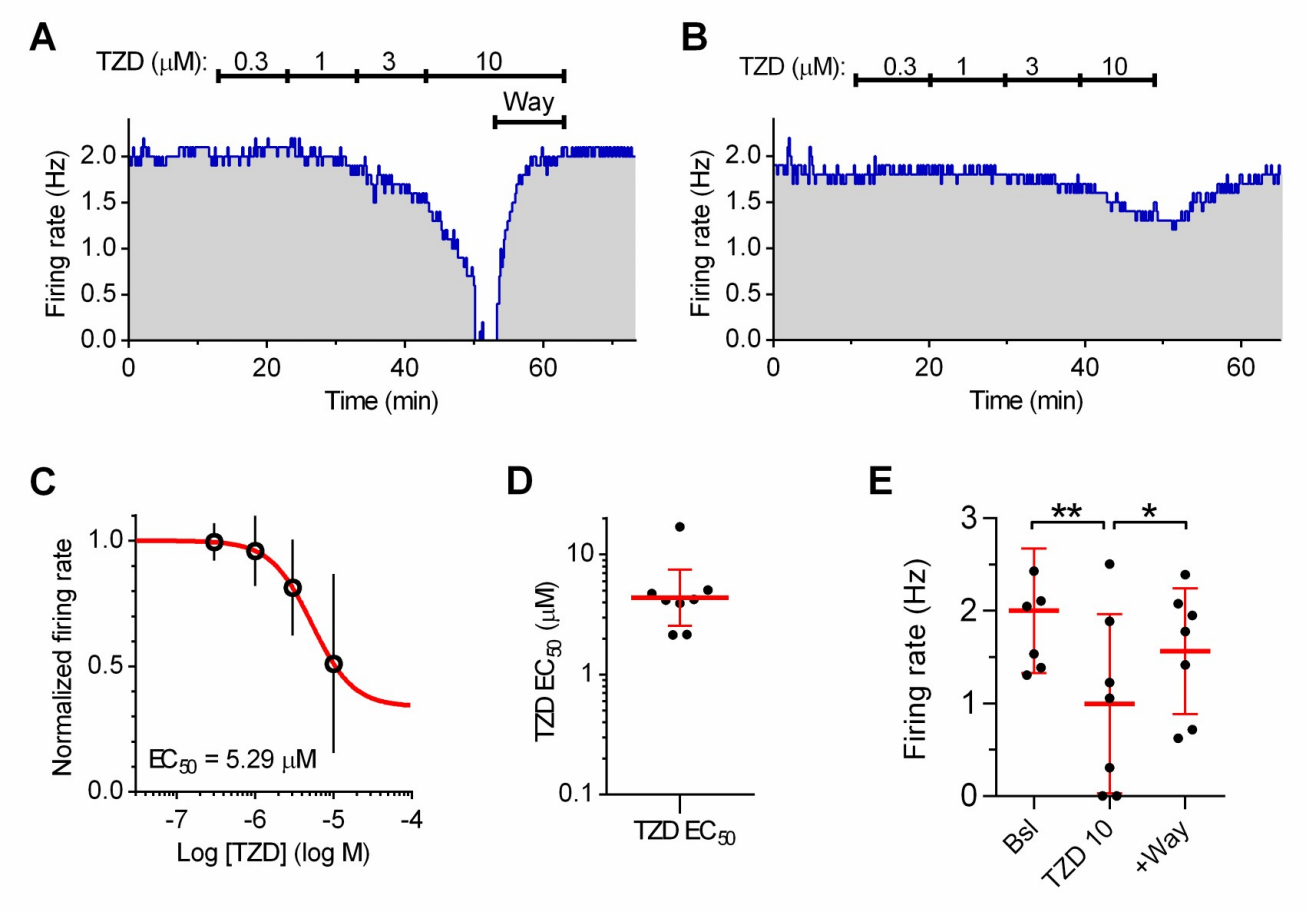
Activation of 5-HT_1A_ receptors by trazodone in high phenylephrine. **(A, B)** Representative recordings of potent **(A)** and weak **(B)** trazodone (TZD effects on serotonergic neuron firing in the presence of 100 μM phenylephrine. Note that trazodone effect in **(A)** was fully antagonized by 20 nM Way-100635 (Way). **(C)** Average DRC obtained from all the experiments (n = 11). **(D)** Summary scatter plot of individual EC_50_ values from experiments in which complete DRC were obtained (n = 8). Data are normalized on Bsl firing rate. Red line is the best least-squares fit to the logistic equation. Symbols and error bars report the mean value ± SD. **(E)** Summary of the antagonism of Way-100635 on trazodone 10 μM (mean ± SD; n = 7; One-way RM ANOVA: F_(2,6)_ = 11.80; p <0.0015). *p< 0.05, **p< 0.01 (One-Way RM ANOVA followed by a Tukey *post-hoc* test).

Based on the experiments shown in Fig 4, in the presence of PE 100 μM the contribution of α_1_-adrenoceptor antagonism in the overall inhibitory effect at concentrations of trazodone up to 10 μM was small (12 ± 8% *see* Fig 4G). We therefore sought to evaluate the potency of trazodone in suppressing serotonergic neuron firing through 5-HT1AAR stimulation in these conditions (i.e. in PE 100 μM) in which the α_1_-adrenoceptor antagonism by the drug was minimized. As shown in Fig 5A and 5B, trazodone inihibited the activity of neurons in a concentration-dependent manner and the effect was completely antagonized by the addition of Way-100635 (20 nM; Fig 5A and 5E).

The average dose-response relationship calculated on the mean response of nine experiments, is reported in Fig 5C. In eight neurons in which individual EC_50_ could be calculated the average EC_50_ value was of 4.365 μM (GM, 95% C.I.: 2.553–7.466, n = 8; Fig 5D). Notably, the curve shown in Fig 5C suggested that the agonism of trazodone at 5-HT_1A_ARs was partial. Nevertheless, due to the residual interference of α_1_-adrenoceptor antagonism by trazodone at concentrations > 10 μM the efficacy of the drug at 5-HT_1A_ARs was impossible to be reliably quantified.

### Trazodone activates 5-HT_1A_ autoreceptor-coupled GIRK channels in serotonergic neurons with weak partial agonist action

**Fig 6:**
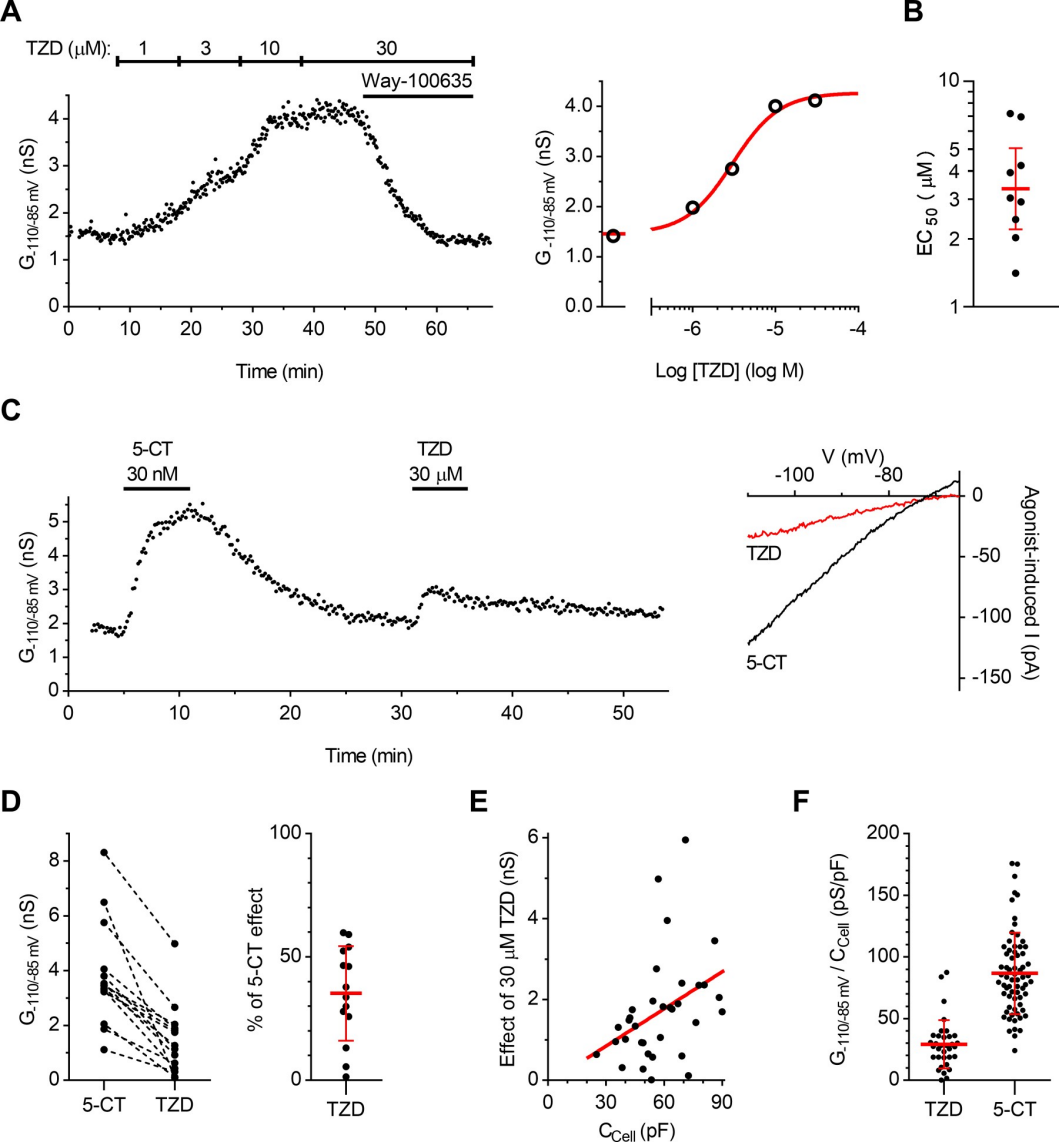
Trazodone partially activates 5-HT_1A_ receptor-coupled GIRK channels of serotonergic neurons. **(A)** Concentration-response relationship for 5-HT1AAR-mediated activation of GIRK conductance by trazodone. *Left panel*: Time-course of a representative experiment showing the effect of increasing concentrations of bath-applied trazodone (TZD) on GIRK conductance (G_-110/-85 mV_) in a serotonergic neuron. G_-110/-85 mV_ was measured as the slope conductance activated in the range from -110 to -85 mV membrane potential by hyperpolarizing ramps under whole-cell voltage-clamp (*see* methods). Application of the selective 5-HT_1A_ receptor antagonist Way-100635 (20 nM) reveals the 5-HT_1A_ receptor-mediated component of the response. Time indicates duration of whole-cell configuration. *Right panel*: concentration-response relationship of the same experiment. The red line is the best least-squares fit to the logistic equation. **(B)** Scatter plot of EC_50_ values of trazodone in individual neurons (n = 9). Note the ordinate in logarithmic scale. Bars correspond to geometric mean ± 95% C.I. **(C)** *Left panel*: Time-course of a representative experiment performed to compare the maximal effect of trazodone (30 μM) with that of the full agonist 5-CT (30 nM) on GIRK conductance (G_-110/-85 mV_) in a serotonergic neuron. *Right panel*: Current-voltage plot of agonist-induced currents of the same experiment. Each trace is the difference between current in the presence of the indicated agonist and control current recorded before the agonist application. **(D)** *Left graph*: Comparison of 30 μM trazodone and 30 nM 5-CT effects in individual neurons. *Right graph*: Scatter plot summarizing the extent of trazodone partial agonism in individual recordings (100 x G_-110/-85 mV, TZD_ / G_-110/-85 mV, 5-CT_). Bars correspond to mean ± SD. **(E)** The correlation between the effect of 30 μM trazodone (G_-110/-85 mV_) and the cell membrane capacitance (C_Cell_). Symbols represent single experiments (n = 34). Red line represents best least square fit. Correlation analysis revealed moderate positive correlation (Pearson *r* = 0.38, p = 0.026). **(F)** Comparison of 30 μM trazodone (n = 34) and 30 nM 5-CT (n = 68) effects in serotonergic neuron population. Data are normalized for cell membrane capacitance. Symbols represent the response of individual neurons to single applications of 5-HT_1A_ receptor agonists. Bars correspond to mean ± SD.

To clarify whether trazodone was a partial agonist, we investigated the potency and the functional effect of trazodone at 5-HT_1A_ARs of rat DRN serotonergic neurons recorded in whole-cell configuration. To this purpose, we measured the changes in slope conductance of 5-HT_1A_ receptor-coupled GIRK channels, which provided a direct measure of 5-HT_1A_ receptor activation produced by the application of trazodone. As illustrated in Fig 6, the concentration-dependent effect of trazodone (1–30 μM; n = 12) was maximal at 30 μM and was reversed by the application of the selective 5-HT_1A_ receptor antagonist Way-100635. In nine out twelve recorded neurons, the magnitude of the concentration-dependent increases in GIRK channel conductance permitted reliable fitting of data in individual concentration-response curves. The average EC_50_ value for GIRK channel opening by trazodone obtained from individual neurons was 3.34 μM (Geometric mean, 95% C.I.: 2.21–5.06; n = 9; Fig 6B).

To quantify the efficacy of trazodone at 5-HT_1A_ARs, we compared the effect of the maximally active concentration of trazodone with that of the 5-HT_1A_ receptor full agonist 5-CT in the same neurons. In all recordings in which 5-CT (30 nM) and trazodone (30 μM) were applied in the same neurons 5-CT produced an increase in GIRK channel conductance greater than that of trazodone (n = 14; Fig 6C) whose effect was occasionally very small (n = 3). From the peak effects produced by trazodone and 5-CT the calculated average efficacy of trazodone compared to 5-CT was 35.3% (Fig 6D; paired t test, two tails: t = 6.588; df = 13; p < 0.0001; n = 14). To confirm these findings in a larger number of neurons and to further ensure that the application of 5-CT in the same neuron did not change the sensitivity to trazodone action at 5-HT_1A_ARs, we compared the response to single applications of 5-CT and trazodone in different neurons. As the magnitude of GIRK conductance responses to trazodone appeared correlated to cell membrane capacitance (Fig 6E), which reflects the surface area of recorded neurons, the effects of trazodone and 5-CT were normalized to the membrane capacitance to correct for differences in cell size and density of GIRK channels in different neurons. The maximal trazodone effect was 29.12 ± 19.63 (pS/pF; n = 34) and resulted significantly smaller compared to that produced by 5-CT (86.68 ± 33.12 pS/pF; n = 68; unpaired t test two tails, t = 9.333; df = 100; p < 0.0001; Fig 6F). The resulting mean efficacy of trazodone was of 33.6% compared to that of 5-CT, a value very similar to that found when the two compounds were applied in the same neurons.

### The weak partial agonism by trazodone partially antagonizes full agonist action of 5-CT at 5-HT_1A_ autoreceptors

**Fig 7:**
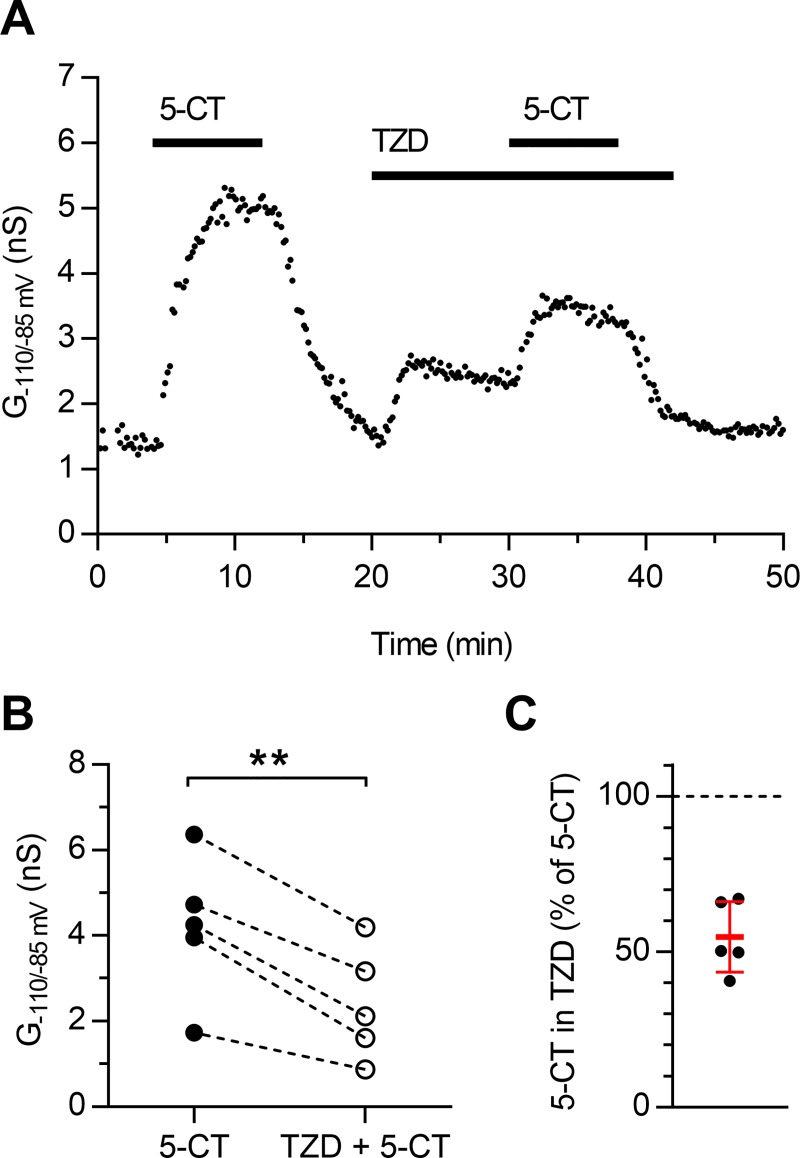
Antagonism at 5-HT_1A_ autoreceptors by trazodone. **(A)** Time-course of a representative experiment in a serotonergic neuron showing that the total activation of the GIRK conductance (G_-110/-85 mV_) produced by 10 nM 5-CT in the presence of 10 μM trazodone (TZD) is smaller than that measured when 5-CT is applied alone. **(B)** Comparison of 10 nM 5-CT effects in control and in the presence of 10 μM trazodone in individual neurons (n = 5). **(C)** Scatter plot summarizing the antagonism of 5-CT effect exerted by trazodone at 5-HT_1A_ARs in five experiments. Values are calculated on net 5-HT_1A_ receptor-activated GIRK conductance obtained from data shown in **(B)** after baseline conductance subtraction. Bars correspond to mean ± SD. **p< 0.01 (paired t test, two tails).

The weak efficacy of trazodone suggested that the drug could exert a competitive antagonism of the response to the full agonist 5-CT. We therefore compared the response to 10 nM 5-CT applied alone or in the presence of 10 μM trazodone. We have chosen submaximal concentrations of the two drugs so that eventual summation of the two effects could be revealed. As illustrated in Fig 7, comparison of peak effects produced by 5-CT alone or in the presence of trazodone (4.21 ± 1.66 vs 2.42 ± 1.29 nS; Fig 7B; n = 5; paired t test, two tails: t = 6.675; df = 4; p = 0.0026) shows that the total activation of the GIRK conductance resulting by coapplication of 5-CT and trazodone was smaller (54.8 ± 11.4%, n = 5, Fig 7C) than that measured when 5-CT was applied alone in the same neurons.

The quantification of the agonist efficacy of trazodone at 5-HT_1A_ARs provided a measured value for a theoretical estimate of 5-HT_1A_AR activation in the absence of trazodone and in the presence of the drug [35] (*see* methods). The calculated response to 10 nM 5 CT in the presence of 10 μM trazodone (42.2% of maximal response produced by 5 CT) was not statistically different from that reported in Fig 7C (54.8 ± 11.4%, n = 5; p = 0.125 Wilcoxon signed rank test).

These results show that trazodone is able to significantly decrease the activation of 5-HT_1A_ARs produced by the full agonist 5-CT and confirmed the applicability of the measured efficacy value to estimate the effects of different trazodone concentrations on the total 5-HT_1A_AR activation by a full agonist using the known affinity constants of the agonists.

## Discussion

Trazodone displays relatively high affinity for several proteins involved in the direct or indirect regulation of serotonergic neuron activity, including 5-HT_1A_ receptors, α_1_-adrenoceptors and SERT [5]. The present study elucidates how the binding of trazodone at these sites translates into direct changes of serotonergic neuron activity. In particular, we characterized the properties of 5-HT1AAR activation by trazodone and the relevance of α_1_-adrenoceptor antagonism in the overall effect of the drug on serotonergic neuron activity. The data reported herein demonstrate that trazodone is an α_1_-adrenoceptor antagonist and a weak partial agonist at 5-HT_1A_ARs of DRN serotonergic neurons. Functionally, these two independent actions converge in inhibiting the activity of serotonergic neurons with variable contribution according to the noradrenergic drive elicited by PE in vitro and, presumably, to the arousal state *in vivo*.

The activity of serotonergic neurons is physiologically facilitated by noradrenergic tone which varies during the sleep-wake cycle, being maximal during wake [36]. At the same time the extracellular 5-HT present in the raphe tonically stimulates 5-HT_1A_ARs thereby limiting neuron firing [37, 38, 12]. The latter action is greatly enhanced when the reuptake of 5-HT is inhibited by antidepressant drugs. This effect is believed to result in a therapeutically inappropriate limitation of serotonergic neuron activity for some weeks, until 5-HT1AAR desensitization occurs [13, 14] or other compensatory mechanisms reconduct serotonergic neuron firing to normal rate in spite of active autoinhibition [39].

Trazodone inhibits the firing of serotonergic neurons recorded from the DRN in anaesthetized rats [19] through an action involving 5-HT_1A_ receptor activation [20]. However, *in vivo* studies did not fully elucidate whether the firing suppression was due to direct activation of 5-HT_1A_ARs, increase in extracellular 5-HT due to SERT inhibition, α_1_-adrenoceptor antagonism, or a combination of these effect. Our experiments show that trazodone directly activates 5-HT_1A_ARs since the effect of the drug was not modified when the local action of the major neurotransmitters was prevented by selective antagonists. Furthermore, the 5-HT_1A_ receptor-mediated inhibition of firing was present in *Tph2*^*-/-*^ mice that lack brain 5-HT indicating that this effect of trazodone was not mediated by endogenous 5-HT acting on serotonergic or neighbour neurons.

In our extracellular recordings the firing of serotonergic neurons was facilitated by the stimulation of α_1_-adrenoceptors with PE to reproduce the activation of serotonergic neurons by noradrenergic drive *in vivo* [40]. Therefore, although in our *in vitro* conditions disfacilitation of firing due to α_1_-adrenoceptor antagonism by trazodone resulted additive to the 5-HT_1A_ receptor-mediated inhibitory effect, these two components of trazodone action could be pharmacologically better discriminated than *in vivo* models. Thus, in the presence of the selective 5-HT_1A_ receptor antagonist Way-100635 the effect of trazodone was concentration-dependently surmounted by increasing PE, indicating that trazodone is a competitive antagonist at α_1_-adrenoceptors. Consistently, when serotonergic neuron firing was facilitated by lowering Ca^2+^ content in the ACSF *in the absence of PE*, trazodone silenced serotonergic neuron firing and the effect was fully antagonized by Way-100635. Furthermore, when the effect of α_1_-adrenoceptor antagonism by trazodone was minimized by the presence of high PE the 5-HT_1A_ receptor-mediated inhibition persisted, although efficacy was much weaker, suggesting that trazodone is a partial agonist at 5-HT_1A_ARs.

The partial agonism of trazodone at 5-HT_1A_ARs was confirmed by means of whole-cell recording in which we quantified the net increase in serotonergic neuron membrane conductance produced by the opening of GIRK channels in response to the drug in the absence of PE. The amount of activated GIRK channels is proportional to the number of receptors stimulated, thus GIRK conductance reliably reports the concentration-dependent effect of agonists allowing for quantitative evaluation of the potency and intrinsic efficacy of trazodone at 5-HT_1A_ARs.

The efficacy of trazodone in activating 5-HT_1A_ARs was about one third of that of the full agonist 5-CT with a potency in the low micromolar range, hence this action would occur at therapeutic concentrations of the drug [41]. The weak efficacy of trazodone in activating 5-HT_1A_ARs is consistent with data obtained using 5-HT_1A_ receptor-stimulated [^35^S]-GTPγS binding in rat hippocampal and cortical membrane preparations (≤ 20% at 30 μM) [21]).

We also show that the weak intrinsic activity of trazodone exerts partial antagonism of the action of the full agonist 5-CT. Thus, at therapeutic concentrations the weak partial agonism of trazodone could antagonize the full activation of 5-HT_1A_ARs of endogenous 5-HT, whose extracellular concentration is raised by the concomitant block of SERT produced by trazodone.

Notably, the partial antagonism exerted by trazodone does not prevent 5-HT1AAR desensitization during treatment *in vivo* (20). This is in agreement with the effect of other partial agonists [42], whereas full antagonists have been shown to prevent functional desensitization of 5-HT_1A_ARs (e.g. Way-100635 [43].

Notwithstanding the more complex effects that trazodone can produce *in vivo* through its action at other receptors (e.g. 5-HT_2A_) or local/long-loop feedback regulation, our data provide the ground for mechanistic interpretation of the interplay between the 5-HT_1A_ receptor agonist and α_1_-adrenoceptor antagonist properties of trazodone in directly regulating serotonergic neuron firing.

Trazodone exerted functionally considerable effects on both 5-HT_1A_ARs and α_1_-adrenoceptors at concentrations ≤ 3 μM that are relevant to the clinical effects of the drug when used as antidepressant or off label hypnotic. One single oral dose of 300 mg trazodone-ER (extended release) produces stable plasma concentrations of ~3 μM for 12 h in healthy volunteers [44]. At this concentration, trazodone would weakly activate 5-HT_1A_ARs but, at the same time, would substantially antagonize the full activation of 5-HT1AARs by 5-HT whose level is raised by the block of SERT produced by the drug. In humans, assuming that the basal extracellular concentration of 5-HT in DRN is similar to that measured in the rat *in vivo* (~10 nM) [45] and that trazodone produces a fivefold increase in 5-HT extracellular level [46]), at therapeutic concentrations achievable in the brain (3 μM) the relative occupancy at 5-HT_1A_ARs by trazodone and 5-HT would be ~70% and ~30% respectively (*see* methods). Under these conditions the total activation of 5-HT_1A_ARs will be ~52% of that produced by 50 nM 5-HT in the absence of the weak partial agonist trazodone.

Interestingly, Ghanbari et al. [20] reported that the basal firing rate *in vivo* after two days of treatment with trazodone was decreased by ~40% compared to controls. This is a relatively weaker inhibition than that observed in similar conditions with escitalopram (~ 70%) [47], although results are from two separate experimental setting, hence not directly comparable.

Importantly, the antagonist effect of trazodone would occur starting from the first drug administration. This would limit the detrimental tonic inhibition of serotonergic neuron firing produced by the raise in extracellular 5-HT starting from the beginning of the therapy, regardless the mechanism(s) involved in the recovery of neuron activity during chronic treatment with antidepressant drugs [13, 14, 39]. Recently, it has been proposed that faster and/or better clinical response to treatment could be achieved with antidepressant drugs that combine SERT inhibition with 5-HT_1A_ receptor partial agonism [18]. In agreement with this hypothesis, clinical signs of antidepressant effect were detected after one week of treatment with trazodone [48]).

Among antidepressant drugs trazodone has a distinctive sleep regulating activity [49]) that favoured its off-label use in insomnia [7]. The 5-HT system participates in arousal and is implicated in sleep, being active during waking and becoming progressively inactive during slow wave sleep and almost completely silent during REM sleep [9, 50, 51, 3]. These state dependent changes in activity of 5-HT system can be ascribed, at least in part, to similar changes in noradrenergic neuron activity [52], that facilitate the firing of serotonergic neurons via activation of α_1_-adrenoceptors [25].

Although the relative contribution of 5-HT_1A_ and α_1_-adrenoceptor effects *in vivo* cannot be fully mimicked *in vitro* as the actual degree of noradrenergic drive changes during wake-sleep cycle, the combined 5-HT_1A_ receptor agonist and α_1_-adrenoceptor antagonist effects of trazodone on these neurons could be relevant to its effects on sleep. At concentrations akin those present in human brain following administration of trazodone for sleep disorders [41], the dual action of trazodone could facilitate inhibition of serotonergic neuron firing in the phase of drowsiness when α_1_-adrenoceptor stimulation is lowered [53]. In addition, antagonism at α_1_-adrenoceptor is likely to dampen the effects of noradrenergic system reactivation that occurs during sleep thereby preventing insomnia-related microarousals [54].

In conclusion, our results show that trazodone directly inhibits DRN serotonergic neuron activity through 5-HT_1A_ receptor weak partial agonism and α_1_-adrenoceptor antagonism. Collectively our data suggest that the overall inhibition of neuron activity produced by the dual action of trazodone will result inversely proportional to the degree of α_1_-adrenoceptor activation, being maximal when α_1_-adrenoceptor is low and gradually decreasing with higher α_1_-adrenoceptor stimulation.
